# Identification and Characterization of Microplastics in Human Cervicovaginal Lavage Fluids Using Raman Spectroscopy: A Preliminary Study

**DOI:** 10.3390/life15030357

**Published:** 2025-02-24

**Authors:** Yoojin Shim, Hyunjin Min

**Affiliations:** 1 Department of Obstetrics and Gynecology, Chung-Ang University Hospital, College of Medicine, 224-1 Heukseok-dong, Dongjak-gu, Seoul 06973, Republic of Korea; yjshimobgy87@cauhs.or.kr; 2 Department of Otorhinolaryngology-Head and Neck Surgery, College of Medicine, Chung-Ang University, 224-1 Heukseok-dong, Dongjak-gu, Seoul 06973, Republic of Korea

**Keywords:** microplastic, human, cervix, vagina, polypropylene, lavage

## Abstract

Microplastics have been detected in various human organs, and studies on their impact on human health are ongoing. However, few studies have researched microplastics in the cervicovaginal area. In this study, we aimed to assess their presence in human cervicovaginal lavage fluid. This prospective study was conducted at a single tertiary medical center, enrolling 10 participants aged 27–49 years. Human cervicovaginal lavage fluid samples were collected from the patients by a single skilled obstetrician. Raman spectroscopy was used to analyze and characterize microplastic particles detected in the samples. Ninety-one microplastic particles were detected in 10 samples. More than 50% of the microplastic particles were identified in a single patient who regularly used menstrual cups. The mean number of microplastics was 9.10 ± 14.96 per 10 g sample. Most of the microplastics were <50 μm in size, and polypropylene and polystyrene were the most predominant types. Raman analysis detected microplastic particles in human cervicovaginal lavage fluids, suggesting that the human cervicovaginal area is exposed to microplastics. The number of detected particles varied significantly among individuals. This study highlights the need for further research on the effects of microplastics on the female reproductive system using cervicovaginal lavage fluid.

## 1. Introduction

Microplastics are small plastic particles measuring less than 5 mm in size, formed either by the fragmentation and degradation of larger plastic debris or by intentional manufacturing for use in a wide range of industrial applications [1]. These particles have become ubiquitous in the natural environment, being detected in soil, oceans, freshwater systems, food, air, and even engineered systems such as wastewater treatment plants. Their widespread presence has raised substantial concerns regarding their potential impact on both human health and ecological systems [2,3]. Microplastics can enter the human body through various pathways, including inhalation of airborne particles, ingestion via contaminated food or water, and dermal exposure during contact with plastic-laden environments [4,5].

Once inside the body, they have been identified in multiple organs, such as the respiratory tract, gastrointestinal tract, and central nervous system, highlighting their pervasive nature and potential to disrupt biological processes [6,7,8,9]. Their accumulation in these vital systems underscores the urgent need for further research into their long-term effects on human health and the environment.

After microplastics were detected in the human placenta, including the maternal, fetal, and amniochorionic membranes, studies have characterized microplastics in the female reproductive system [10]. Recently, microplastics have been identified in adenomyosis, ectopic ovarian cysts, and uterine tubes [11]. Similarly, microplastics in the human endometrium have been identified, and an invasion model has been suggested [12]. Xu et al. [13] reported microplastic particles in human uterine fibroids and myometrium tissues, demonstrating that microplastics are more abundant in uterine fibroids than in myometrium tissues. The toxicity of microplastics in the reproductive system has been reported in previous studies based on animal experiments [14,15,16]. The lower part of the uterus is connected to the vagina by the lower section of the cervix, while the upper section of the cervix connects to the upper part of the uterine cavity through the endocervical canal, which is linked to the endometrium [17]. This anatomical connection leads to the presence of microorganisms, metabolites, and immune components from X in the female reproductive tract microenvironment. The balance of the interactions among these factors is important for maintaining female reproductive tract homeostasis and health [18].

From this anatomical perspective, we focused on how microplastics present in the female reproductive system may have entered the cervix from external sources. As little research has been conducted on microplastics in the cervicovaginal area, we investigated whether microplastics are present in this area. Based on a previous study that detected microplastics in fluids obtained from human nasal samples [7], this current preliminary study collected cervicovaginal lavage fluid samples to determine whether microplastics were present in the cervicovaginal region.

## 2. Materials and Methods

### 2.1. Sample Preparation

This prospective study was conducted at a single tertiary medical center between June 2024 and September 2024. The research was conducted in accordance with the Declaration of Helsinki and approved by the Institutional Review Board (IRB) of Chung-Ang University Hospital (IRB number: 2405-003-600, 28 June 2024). Written informed consent was obtained from all study participants for performing cervicovaginal lavage and collecting the fluid prior to the start of surgery.

Samples were obtained from 10 patients who underwent hysteroscopy using a resecto-hysteroscope for thickened endometrium and suspected endometrial polyps. The study excluded women with pregnancy, active vaginal bleeding, genital infection, cervical pathology, or other concurrent immunomodulatory medical diseases, including malignancy and autoimmune diseases. Medical records, including clinical characteristics, smoking history, and underlying medical diseases, were further reviewed and analyzed.

Cervicovaginal lavage fluid was collected from the patients by a skilled obstetrician. Under general anesthesia, patients were placed in the lithotomy position. The obstetrician inserted a speculum without using a lubricant and applied a resecto-hysteroscope with an outer sheath of 8 mm, with a 30-degree grade optic (Olympus, Tokyo, Japan). The control sample was collected by passing a 0.9% saline solution only through a hysteroscope. The experimental samples were collected by passing a 0.9% saline solution through the hysteroscope and the cervicovaginal area of the patient for irrigation. We used a metal suction (tube/device) and glass bottles to collect approximately 20 mL of fluid before proceeding with surgery. In this study, no additional pretreatment was applied to the cervicovaginal fluid during sample collection. Ten grams of each harvested fluid was stirred (100 revolutions/min) with 50 mL of 30% H_2_O_2_ for 7 days at 60 °C. The samples were then filtered through a 5 μm silicon filter (10 × 10 mm^2^; SmartMembranes, Halle, Germany) to collect microplastics for subsequent analysis.

### 2.2. Raman Spectroscopy

Raman analysis was conducted using an XploRA Plus Confocal Raman microscope (HORIBA France SAS, Longjumeau, France) [7,19] equipped with a 532 nm laser and a cooled charge-coupled device detector with a resolution of 1024 × 256 pixels. The system incorporated an embedded filter to reduce the laser power by 10%, ensuring optimal sample safety and data accuracy. Gratings with 1200 grooves/mm were employed to achieve precise spectral resolution. The confocal hole and slit widths were adjusted to 100 μm and 50 μm, respectively, to optimize the spatial resolution and minimize interference. Calibration of the system was carried out by performing zero-order correction of the grating and referencing the characteristic silicon peak at 520.7 cm⁻^1^.

Mosaic microscopic dark-field images of the filter surfaces from the samples were captured using an M Plan Semi-Apochromat BD objective lens (20×/N.A. 0.45, Olympus, Tokyo, Japan). These images were processed using the ParticleFinder module within the LabSpec 6 software, which facilitated the identification of bright particles against a dark background for subsequent Raman analysis. Raman spectra were recorded across a spectral range of 1020–3400 cm⁻^1^, with an exposure time of 1 × 2 s for each measurement.

**Figure 1:**
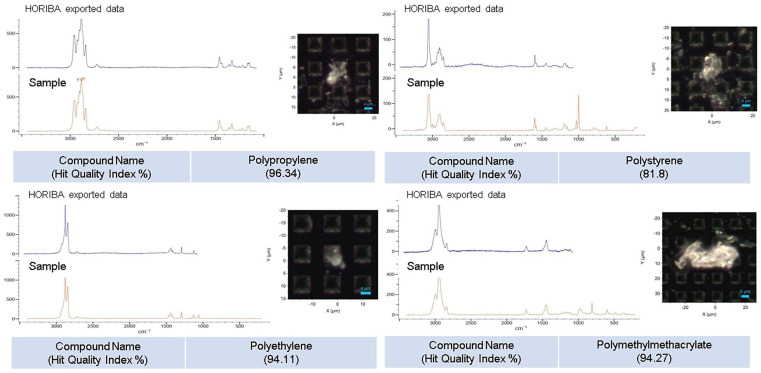
Examples of microphotographs and Raman analysis of major microplastic types.

The collected spectra underwent baseline correction using a polynomial method to remove background interference. Spectra with significant fluorescence were excluded to ensure data reliability. The classical least-squares (CLS) algorithm was applied to screen all spectra for plastic identification. The standard reference spectra used in the CLS fitting process included polypropylene, polyethylene, polystyrene, polyethylene terephthalate (PET), and polymethyl methacrylate (PMMA) (Figure 1). The spectra for each sample were derived by summing the contributions of the reference polymers and the theoretical compositions. A manual review of the CLS fitting results was conducted to prevent errors such as false positives or omissions, which could arise from automated spectrum matching against the Raman library.

### 2.3. Quality Control

Plastic materials were excluded from the experiments to minimize the risk of microplastic contamination. Only glass materials were used throughout the sampling and filtering processes. All glassware was rinsed with filtered ultrapure water before the experiments. All procedures, including sample preparation and filtration, were performed in a laminar flow hood (HSCV-1300; SINAN Science Industry, Beijing, China) to prevent airborne microplastic contamination. All solutions, including ultrapure water and chemical reagents, were pre-filtered using glass fiber filters (grade F, medium, high loading) and metal filters (0.5 μm) before use. The samples were wrapped in aluminum foil during transfer outside the laminar flow hood. To minimize the risk of contamination, we wore nitrile gloves and cotton lab coats throughout processing. Three blank samples were prepared using empty glass beakers and subjected to the same procedure. The obtained results were compared with those from the experimental groups to evaluate potential contamination during sample pretreatment and analysis.

## 3. Results

### 3.1. Number of Microplastics in Cervicovaginal Lavage Fluids

The demographic characteristics of the enrolled participants are shown in Table 1. The mean age of the 10 participants was 38.60 ± 7.05 (27–49) years. One participant each was diagnosed with myoma, adenomyosis, and hypothyroidism; however, no other systemic diseases that could potentially influence the study results were present. Nine patients were diagnosed with endometrial polyps, and one was diagnosed with atypical endometrial hyperplasia. During the menstrual period, nine patients used sanitary pads, and one regularly used a menstrual cup (Table 1).

Raman spectroscopy was used to analyze a total of 10 cervicovaginal lavage fluid samples obtained from the participants (Figure 1). Detailed results regarding the number of microplastics found in each sample are summarized in Table 2. One microplastic was detected in the laboratory control sample (blank), whereas no microplastics were identified in the control fluid collected without contact with the cervix. A total of 91 microplastics were detected in the cervicovaginal lavage fluid samples. The mean concentration of microplastics was 9.10 ± 14.96 particles per 10 g of the sample. More than half of the detected microplastics were concentrated in a single sample belonging to Patient 4, who used a menstrual cup until shortly before surgery (51 microplastics per 10 g, Table 1). When the data from Patient 4 were excluded, the adjusted mean count of microplastics in the remaining samples decreased significantly to 4.44 ± 2.83 particles per 10 g. This suggests a potential link between the type of menstrual product used and microplastic contamination in cervicovaginal lavage fluid.

### 3.2. Characterization of the Microplastics in the Cervicovaginal Lavage Fluid Samples

**Figure 2:**
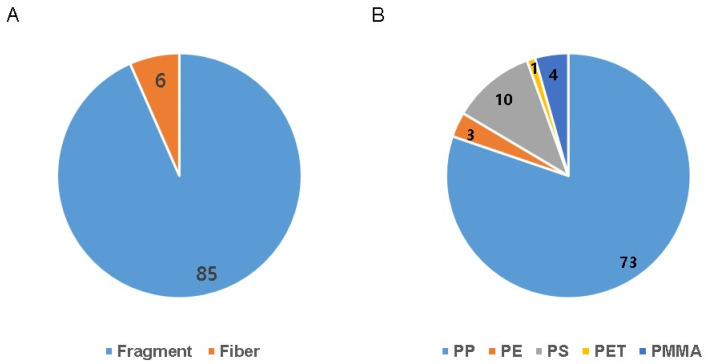
(**A**) Shape and (**B**) polymer types of the 91 microplastic particles detected in human cervicovaginal lavage fluids. PE: polyethylene; PET: polyethylene terephthalate; PMMA: polymethylmethacrylate; PP: polypropylene; PS: polystyrene.

The detected microplastics were characterized according to their shape, size, and polymeric composition. In terms of shape, the predominant category consisted of fragment-like particles (n = 85, 93.4%), while fiber-like particles accounted for a smaller proportion (n = 6, 6.6%; Figure 2A). The size distribution analysis revealed that microplastics within the 5–10 μm range were the most frequently detected, followed by microplastics measuring 10–20 μm in size (Table 2). Microplastics larger than 50 μm were found less frequently. The polymer composition analysis identified five distinct types of materials: polypropylene, polyethylene, polystyrene, PET, and PMMA (Figure 2B). Among these, polypropylene (n = 73, 80.2%) and polystyrene (n = 10, 11%) were the most commonly identified polymers in the cervicovaginal lavage samples.

## 4. Discussion

In this study, we aimed to determine whether microplastics could be detected in human cervicovaginal lavage fluids. Our results indicate that microplastics in the female reproductive system could be detected and characterized from the organ’s surface fluid, represented as cervicovaginal lavage fluid, instead of direct tissue samples. More than 50% of the microplastic particles were identified in a single patient, who was the only one who regularly used menstrual cups. Considering that the cervicovaginal lavage fluid samples were collected by a single skilled surgeon using the same protocol over a relatively short period, the concentration of microplastics detected in cervicovaginal lavage fluids was influenced by patient behavior rather than the collection method.

The mean number of microplastics was 9.10 ± 14.96 per 10 g sample, and most microplastics were 5–10 μm of the polypropylene type. A recent study based on 60 patients who underwent surgical removal of adenomyosis, ovarian ectopic cysts, and uterine tubes found an average microplastic concentration of 1.40 ± 1.11 particles per g of tissue; 70% of the microplastics were <20 μm, with polyethylene and polypropylene being the most common types [11]. These findings are similar to ours, although we used organ surface irrigation fluids and did not investigate human tissue itself.

One microplastic particle was detected in the blank sample, one in a cervicovaginal lavage fluid sample (Patient 8), and two samples had two microplastics (Patients 2 and 3). Excluding Patient 4, the number of microplastics averaged 4.44 ± 2.83 per 10 g. We cannot be confident that the study results (the difference between the sample and blank) were clinically significant. However, previous studies have reported that the concentration of microplastics detected in the female reproductive system is very low. For example, a total of 12 microplastic particles were detected in the placentas of four women [10]; in a different study, microplastics were detected in only 72% of the 60 tissues, with a total of 90 microplastic particles in 60 human reproductive organ tissues [9]. Another study found 2.13 ± 1.17 particles/g in 16 human uterine fibroids and 0.88 ± 0.78 microplastic particles/g in eight myometrium tissues [13].

As our results based on surface irrigation fluids demonstrated a similar microplastic particle density to those reported based on tissues in the female reproductive system, we emphasize the need for further research on possible differences in microplastic concentration between tissue and cervicovaginal lavage fluids obtained from the tissue surface. Ou et al. [20] reported the metabolic fingerprints of cervicovaginal lavage using liquid chromatography-mass spectrometry; however, we found no studies evaluating microplastics in indirect samples, as carried out in our study. Furthermore, no study has measured and analyzed microplastics simultaneously in tissue and irrigation fluid from the same female reproductive system organ. Such research is expected to establish a standard for obtaining samples less invasively, enabling rapid results without preprocessing.

Microplastics enter the human body through direct absorption routes, including inhalation, skin contact, and ingestion. After entering the body, they travel through the bloodstream or penetrate tissues directly, reaching different organs. For example, microplastics ingested through diet and water may migrate through the gastrointestinal tract, penetrate the tissue barrier, and accumulate in tissues and organs [12,21,22]. For the female reproductive system, experiments in mice demonstrated that the invasion of the uterus by microplastics was modulated either through diet–blood circulation or via a vagina–uterine lacuna model [12]. Our study determined that recent use of a menstrual cup resulted in the detection of many microplastics. Considering that microplastics can penetrate the human body through products commonly used by women (such as sanitary pads, cups, or tampons), large population-based studies involving a broader age range are needed to identify the sources and invasion mechanisms of microplastics in the female reproductive system.

Accumulation of microplastics causes inflammation, oxidative stress, and immune responses, resulting in various health problems in humans [13,23,24]. Microplastics can also cause reproductive and developmental health problems. In male mice, polystyrene microplastics decrease sperm concentration, motility, and proportion of normal sperm, suggesting reproductive dysfunction [25]. Co-exposure to polystyrene microplastics and di-2-ethylhexylphthalate activated the TGF-β1/Smad3 signaling pathway; inhibiting this pathway alleviates oxidative stress, hormonal dysregulation, and ovarian fibrosis in female rats [26]. Microplastics can penetrate the placental barrier, interfere with offspring development, have cross-generational effects, and cause reproductive toxicity and genotoxic effects [13,27,28]. Although studies on microplastics in the female reproductive system and microplastic toxicity have been reported for in vitro and in vivo experiments, comprehensive research connecting the two to assess the effect of microplastics on adverse fertility or pregnancy outcomes in humans is necessary [27].

This study has a few limitations. The results were obtained from a relatively small cohort of patients, which may limit the generalizability of the findings to a broader population. The limited number of participants also made it difficult to draw conclusions through statistical analysis. Additionally, this study served as a preliminary investigation aimed at detecting the presence of microplastics specifically in cervicovaginal lavage fluid rather than in tissue samples. While our identification and characterization of microplastics in these fluid samples provide valuable insights, the findings underscore the need for follow-up studies involving larger sample sizes to confirm and expand upon these results. Moreover, maintaining the quality of controls is a crucial aspect of microplastic analysis. In this study, the microplastics detected in the controls are believed to result from partial contamination during the cervicovaginal sampling process. Establishing a sampling protocol to address these issues is an important research topic. Another limitation is the size detection threshold of our analytical method, which was restricted to particles larger than 5 µm. We identified only microplastics that exceeded this size limit. However, nanoplastics—particles smaller than microplastics—are also of significant concern because of their potential harmful effects on the reproductive system. These smaller particles can directly damage cellular structures or disrupt genetic pathways, particularly those associated with the hypothalamic–pituitary–gonadal axis [28,29,30]. Future studies should prioritize evaluating the presence and effects of nanoplastics in female reproductive organs to address this gap. Finally, we could not control all the factors that could have influenced the study outcomes, such as variations in patients’ living environments and dietary habits. Although meals were provided on a standardized schedule from admission until the day before surgery, detailed information about the patients’ long-term dietary patterns and environmental exposure was lacking. These factors could contribute to the presence of microplastics in the body, even in organs that are not directly connected to the gastrointestinal tract. Moving forward, it will be crucial to incorporate comprehensive questionnaires or other tools to assess how microplastics might enter the body through food consumption or environmental contact, thus enabling a more thorough understanding of their impact.

## 5. Conclusions

This study demonstrated that microplastics can be detected in human cervicovaginal lavage fluids using Raman spectroscopy. In addition, the analysis of the number and characteristics of the identified microplastics showed that the findings were similar to previously reported results obtained from female reproductive organs, including the uterus and ovaries. The results of this study indicate the need for further research on the effects of microplastics on the female reproductive system using cervicovaginal lavage fluid. Further investigations into measuring microplastics in the female reproductive system and evaluating their potential effects on human health are necessary.

## Figures and Tables

**Table 1:** Characteristics of the enrolled patients.

Patient	Age (Years)	BMI (kg/m^2^)	Concurrent Diagnosis	Reason for Hysteroscope	Menstrual Product Used	Duration of Menstrual Product Usage (Days/Month)
1	46	28.55	None	Endometrial polyp	Sanitary pads	7.5
2	45	20.96	Myoma	Endometrial polyp	Sanitary pads	7
3	34	36.65	None	Endometrial polyp	Sanitary pads	6.4
4	38	23.31	None	Endometrial polyp	Menstrual cups	7
5	49	20.83	None	Endometrial polyp	Sanitary pads	10
6	38	21.71	Adenomyosis	Endometrial polyp	Sanitary pads	7
7	42	34.67	None	Atypical endometrial hyperplasia	Sanitary pads	7
8	37	21.67	Hypothyroidism	Endometrial polyp	Sanitary pads	10
9	27	21.18	None	Endometrial polyp	Sanitary pads	20
10	30	29.07	None	Endometrial polyp	Sanitary pads	7

BMI: body mass index.

**Table 2:** Number of microplastics in cervicovaginal lavage fluids according to size range.

Sample	Microplastic Particles According to Size Length (μm)					Total Particles
	5–10	10–20	20–50	50–100	>100	
Blank	1	-	-	-	-	1
Control	-	-	-	-	-	0
Patient 1	2	-	-	-	-	2
Patient 2	-	1	-	1	-	2
Patient 3	2	3	4	-	-	9
Patient 4	28	16	7	-	-	51
Patient 5	2	2	2	-	-	6
Patient 6	-	1	2	-	-	3
Patient 7	1	0	2	-	-	3
Patient 8	0	1	0	0	0	1
Patient 9	3	2	2	0	2	7
Patient 10	5	2	-	-	-	7

## Data Availability

Data available on request due to privacy/ethical restrictions.

## Abbreviations

The following abbreviations are used in this manuscript:

CLS	classical least-squares
PET	polyethylene terephthalate
PMMA	polymethylmethacrylate
